# Immunity, Inflammation and Heart Failure: Their Role on Cardiac Function and Iron Status

**DOI:** 10.3389/fimmu.2019.02315

**Published:** 2019-10-01

**Authors:** Maria Perticone, Roberta Zito, Sofia Miceli, Angelina Pinto, Edoardo Suraci, Marta Greco, Simona Gigliotti, Marta Letizia Hribal, Salvatore Corrao, Giorgio Sesti, Francesco Perticone

**Affiliations:** ^1^Department of Experimental and Clinical Medicine, University Magna Graecia of Catanzaro, Catanzaro, Italy; ^2^Department of Medical and Surgical Sciences, University Magna Graecia of Catanzaro, Catanzaro, Italy; ^3^Department of Geriatrics, Azienda Ospedaliero-Universitaria Mater Domini, Catanzaro, Italy; ^4^Department of Health Sciences, University Magna Graecia of Catanzaro, Catanzaro, Italy; ^5^Department of Internal Medicine 2, National Relevance and High Specialization Hospital Trust, Palermo, Italy

**Keywords:** ejection fraction, heart failure, iron deficiency, toll-like receptor, inflammation

## Abstract

**Aims:** Heart failure is a clinical syndrome characterized by subclinical systemic inflammation and immune system activation associated with iron deficiency. No data exist on the various activations of immune-mediated mechanisms of inflammation in heart failure patients with reduced/preserved ejection fraction. We aimed to (1) investigate possible differences in inflammatory parameters and oxidative stress, and (2) detect a different iron status between groups.

**Materials and Methods:** We enrolled 50 consecutive Caucasian outpatients with heart failure. All patients underwent echocardiographic measurements, laboratory determinations, evaluation of iron status and Toll-like receptors, and NF-κB expression in peripheral blood mononuclear cells, as well as pro-inflammatory cytokines. All statistical calculations were made using SPSS for Mac version 21.0.

**Results:** Patients with reduced ejection fraction showed significantly lower hemoglobin levels (12.3 ± 1.4 vs. 13.6 ± 1.4 g/dl), serum iron (61.4 ± 18.3 vs. 93.7 ± 33.7 mcg/dl), transferrin iron binding capacity (20.7 ± 8.4 vs. 31.1 ± 15.6 %), and e-GFR values (78.1 ± 36.1 vs. 118.1 ± 33.9 ml/min/1.73 m^2^) in comparison to patients with preserved ejection fraction, while unsaturated iron binding capacity (272.6 ± 74.9 vs. 221.7 ± 61.4 mcg/dl), hepcidin (4.61 ± 0.89 vs. 3.28 ± 0.69 ng/ml), and creatinine (1.34 ± 0.55 vs. 1.03 ± 0.25 mg/dl) were significantly higher in the same group. When considering inflammatory parameters, patients with reduced ejection fraction showed significantly higher expression of both Toll-like receptors-2 (1.90 ± 0.97 vs. 1.25 ± 0.76 MFI) and Toll-like receptors-4 (4.54 ± 1.32 vs. 3.38 ± 1.62 MFI), respectively, as well as a significantly higher activity of NF-κB (2.67 ± 0.60 vs. 1.07 ± 0.30). Furthermore, pro-inflammatory cytokines, interleukin-1, and interleukin-6, was significantly higher in patients with reduced ejection fraction, while the protective cytokine interleukin-10 was significantly lower in the same group. Correlational analyses demonstrated a significant and inverse relationship between left ventricular function and inflammatory parameters in patients with reduced ejection fraction, as well as a direct correlation between ferritin and inflammatory parameters.

**Conclusions:** Our data demonstrate a different immune-mediated inflammatory burden in heart failure patients with reduced or preserved ejection fraction, as well as significant differences in iron status. These data contribute to further elucidate pathophysiologic mechanisms leading to cardiac dysfunction.

## Introduction

Heart failure (HF) is a complex clinical syndrome, characterized by impairment of both the cardiac structure and function leading to left ventricle filling or ejection abnormalities. In addition, HF is one of the most common causes of hospitalization recurrence and death (1, 2). Recently, according to the value of echocardiographic left ventricular ejection fraction (EF), HF guidelines have identified three main forms of HF including: HF with reduced EF (HF-rEF; EF ≤40%), HF with preserved EF (HF-pEF; EF ≥50%), and an intermediate form with an EF ranging from 41 to 49% (1).

**Figure 1:**
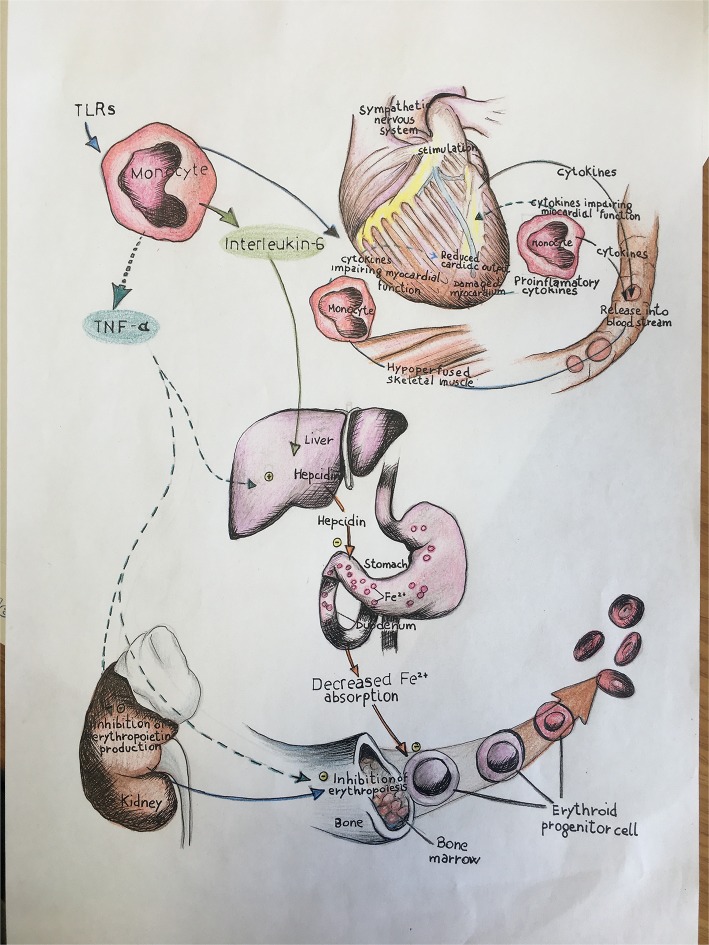
In this illustration we schematically report the role of immune-mediated inflammation in iron status balance and the consequent effects on cardiac function in heart failure. TLRs, toll-like receptors; TNF-α, tumor necrosis factor alpha; IL, interleukin.

It is well established that HF patients, independent from prevalent systolic or diastolic dysfunction, show increased levels of pro-inflammatory cytokines, associated to adverse clinical outcomes (3, 4). This inflammatory status, probably driven by the coexistence of traditional cardiovascular (CV) risk factors, is maintained by other mechanisms such as immune system activation (3, 4). In addition, this chronic subclinical systemic inflammatory status is associated with iron deficiency (ID), even in absence of clinically evident anemia (5, 6), which also contributes to HF progression and associated mortality (7) (Figure 1). Randomized trials (8–10) of intravenous ferric carboxymaltose in the treatment of ID in HF-rEF patients demonstrated an improvement of symptoms, and this therapeutic approach should be considered when treating ID in this setting of patients, in order to alleviate symptoms.

The subclinical inflammation seen in HF patients is due to the higher activation and expression of toll-like receptors (TLRs), in particular TLR-2 and TLR-4 that, in turn, induces the gene transcription of pro-inflammatory cytokines (11–13).

To our knowledge, at this time, no data exist on a different activation of the immune-mediated mechanisms of inflammation in HF-rEF and HF-pEF patients. Thus, this study is aimed to: (1) investigate possible differences in inflammatory parameters and oxidative stress [evaluated by nuclear factor kB (NF-κB)] between HF-rEF and HF-pEF patients, and (2) to detect a different iron status in the two groups.

## Materials and Methods

In this study we enrolled 50 consecutive Caucasian outpatients, 34 males and 16 females with a mean age of 70.7 ± 10.7 years. HF diagnosis was carried out according to the recent guidelines of the European Society of Cardiology (6), considering the presence of symptoms and/or signs, as well as echocardiographic parameters. For HF-pEF echocardiographic parameters considered were left ventricular (LV) EF ≥50 %, and evidence of LV diastolic dysfunction. For HF-rEF echocardiographic parameters considered were LV volumes, a LVEF of 40% or less, LV wall thickness, functioning valves, and pulmonary hypertension. Exclusion criteria were history of autoimmune diseases and all the conditions associated with immune system activation, hemochromatosis, and other conditions associated with iron metabolism disorders, other forms of anemia different from the sideropenic one, cardiac storage diseases, congenital cardiomyopathies, myocarditis or dilated cardiomyopathy myocarditis-related, malignancies, thyroid dysfunctions, liver insufficiency, and administration of any drug interfering with iron metabolism (iron supplementation, antibiotics, diuretics, laxatives, fibrates, antacids, proton pump inhibitors, metformin, biguanides, alcohol). NYHA classification was performed according to current guidelines. The Ethical Committee approved the protocol and informed written consent was obtained from all participants. All the investigations were performed in accordance with the principles of the Declaration of Helsinki.

### Echocardiographic Measurements

Tracings were taken 24–48 h after laboratory/clinical determinations with patients in a partial left decubitus position, using a VIVID-7 Pro ultrasound machine (GE Technologies, Milwaukee, WI) with an annular phased array 2.5 MHz transducer. Echocardiographic readings were taken in a random order by the investigator, who had no knowledge of patients' blood pressure (BP) and other clinical data. Only frames with optimal visualization of cardiac structures were considered for the reading. The mean values from at least five measurements of each parameter for each patient were computed. Having the same experienced sonographer (MS) performing all studies in a dimly lit and quiet room, we optimized the measurements reproducibility. In our laboratory, the coefficient of variations was 3.35% for posterior wall (PW) thickness, 3.40% for inter-ventricular septum (IVS) thickness, 1.30% for left ventricular internal diameter (LVID), and 4.10% for left ventricular mass (LVM). Tracings were recorded under two-dimensional guidance, and M-mode measurements were taken at the tip of the mitral valve or just below. Measurements of IVS, thickness, PW thickness, and LVID were made at end-diastole and end- systole. LVM was calculated using the Devereux equation (14) and normalized by body surface area (i-LVM).

Measurements of EF were obtained by Simpson method. LV diastolic function was evaluated according to diagnostic criteria proposed by the American Society of Echocardiography (15). Evaluation of left atrium volume, indexed for body surface area (i-LAV), was obtained using the apical four-chamber and two-chamber views. Pulsed Doppler trans-mitral flow velocity profile was obtained from the apical four-chamber view, and the sample volume was located at the tip of the mitral valve leaflets. The following parameters were evaluated for diastolic function: peak trans-valvular flow velocity in early diastole (E wave), peak trans-valvular flow velocity in late diastole (A wave), E-to-A ratio. Tricuspid regurgitant velocity (TRV), a useful parameter to calculate systolic pulmonary arterial pressure (s-PAP) and pulmonary vascular resistances (PVR), was derived from the application of continuous wave Doppler along the tricuspid regurgitant jet, using an apical four chamber projection or a parasternal right ventricle (RV) inflow view for eccentric regurgitant jet (16). s-PAP was calculated through the Bernoulli equation: s-PAP = 4 (TRV)^2^+PVR. PVR were calculated using the following formula: TRV peak/RVOT time velocity integral (TVI) x10+0.16 (17).

### Laboratory Determinations

All laboratory measurements were performed after fasting for 12 h. Plasma glucose was determined immediately by the glucose oxidation method [Glucose analyzer, Beckman Coulter, Milan; intra-assay coefficient of variation (CV) 2.2%, inter-assay CV 3.8%]. Total, low-density lipoprotein- (LDL), and high-density lipoprotein- (HDL) cholesterol and triglyceride concentrations were measured using enzymatic methods (Roche Diagnostics GmbH, Mannheim, Germany). Uric acid and creatinine were measured using the Jaffe methodology. Values of estimated glomerular filtration rate (eGFR) (ml/min/1.73 m^2^) were calculated using the CKD-EPI equation. We preferred this equation because it was developed from a much larger cohort of patients, including both normal individuals and those with chronic kidney disease. High-sensitivity C-reactive protein (hs-CRP) levels were measured with an automated instrument (Cardio- Phase hs-CRP, Siemens Healthcare). Fibrinogen is dosed with a coagulation method on the instrument BCSXP. The complete blood count was performed by flux cytometry.

### Iron Status Evaluation

According to current recommendations (18), we defined ID as: ferritin <100 μg/dl or ferritin of 100–299 μg/dl with a transferrin saturation <20%; we adopted this definition because pathogenetic mechanisms underlying iron abnormalities in HF differ from other conditions of chronic inflammation. According to this we also evaluated hepcidin concentration.

Serum iron was evaluated by a colorimetric test and direct determination with ferro-zine; unsaturated iron binding capacity (UIBC) and the percentage of saturated transferrin iron binding capacity (TIBC) were measured using a colorimetric test (Roche Diagnostics, Cobas 8000, Switzerland). Ferritin levels were measured using an immunoturbidimetric assay (Roche Diagnostics, Indianapolis, IN, USA). Transferrin values were evaluated by nephelometric method.

### Serum Hepcidin Evaluation

Serum hepcidin concentration was assessed with a specific ELISA kit (Abbexa Human Hepcidin 25; Catalog No abx350221), according to the manufacturer's instructions. The standards and samples are added to the wells, incubated and washed with wash buffer. A biotin conjugated antibody specific to hepcidin 25 is used for detection. TMB substrate is used to visualize HRP activity. TMB is catalyzed by HRP to produce a blue colored product that changes into yellow after adding stop solution. O.D. absorbance was measured spectrophotometrically at 450 nm in a microplate reader, and the concentration of Hepc25 was then calculated as the variation over the control. Intra and inter assay precision values were <10%, respectively.

### Evaluation of TLR2 and TLR4 Expression

Peripheral blood mononuclear cells (PBMCs) were isolated from fresh peripheral samples by Ficoll gradient according to the manufacturer's instructions within 2 h after blood collection. Isolated PBMCs were harvested and suspended in phosphate buffered saline (PBS) for TLRs expression analysis by FACS, or in hypotonic buffer and further processed to evaluate NF-κB (p65) activity. Isolated PBMCs were labeled with an anti-CD14 antibody conjugated with phycoerythrin (PE) to identify the monocytes within the isolated population and with an anti-TLR-2 antibody conjugated with fluorescein isothiocyanate (FICT), or with an anti-TLR-4 antibody conjugated with allophycocyanin (APC). An ISO CD-14 (PE) non-specific antibody was employed as a control isotype to exclude non-specific bonds. Samples were acquired with a FACSCalibur (BD Biosciences) and analyzed by Flow JO software (LLC). The monocyte population was identified by both forward (FSC) and side scatter (SSC). TLRs expression was considered as the ratio of mean fluorescence intensity (MFI) of the sample and MFI of the isotype control.

### NF-κB(p65) Activity Assay

Successively, to evaluate NF-κB(p65) activity PBMCs were lysed with a hypotonic buffer containing 10 mM Hepes, 0.1 mM EDTA and phosphatase inhibitors. Supernatants containing cytosolic fractions were removed and pellets were resuspended in a nuclear extraction buffer containing 10 mM Hepes, 0.1 mM EDTA, 1.5 mM MgCl_2_, 420 mM NaCl, and 10% glycerol. NF-κB activity in nuclear lysates was then measured with NF-κB specific ELISA assay (Cayman Chemical Company, Ann Arbor, MI, USA), according to the manufacturer's instructions. A positive control (human p65 transcription factor *NF-*κ*B*) was included in each ELISA plate; samples were assessed in duplicate.

### Pro-inflammatory Cytokines Evaluation

To assess cytokine levels, serum samples were loaded into a biochip array containing specific primary antibodies (Cytokine and Growth Factors Array, Randox Laboratories) and processed according to the user manual. Briefly, biochips were incubated for 1 h at 37°C, washed and incubated for an additional hour with the HRP-conjugated secondary antibody. Biochips were acquired with the biochip reader Evidence Investigator (Randox Labs, UK) and the “Cytokines Array I and High sensitivity” kit, Randox UK, quantified with its dedicated software. The assay measuring range was 0–250 pg/ml for IL-6; 0–900 pg/ml for IL-8; 0–400 pg/ml for IL-10; 0–600 for tumor necrosis factor (TNF)-α, and 0–600 pg/ml for monocyte chemoattractant protein (MCP)-1. Intra and inter assay precision values were 7.3 and 12.9 %, respectively, for IL-6; 7.7 and 6.7% for IL-8; 7.8 and 5.2% for IL-10; 9.9% for TNF-α; 3 and 7.6% for MCP-1.

### Statistical Analysis

Descriptive data are presented as the mean ± SD for normally distributed variables, and binary data as percent frequency. Distribution normality was assessed with the Shapiro–Wilk test. Groups were compared using an unpaired *t*-test when clinical and biological data were expressed as continuous variables and the χ^2^ test for categorical variables. Correlational analyses were performed using the Pearson's test. Differences were assumed to be significant at two-tailed *P*-values < 0.05. All calculations were done with a standard statistical package (SPSS for Mac version 21.0, Chicago, IL, USA).

## Results

On the basis of their EF, patients were divided into HF-rEF and HF-pEF groups. All patients were in the NYHA II-III functional class.

In Table 1 we reported the anthropometric, biochemical, and hemodynamic characteristics of the whole study population and of the two groups separately. The mean NHYA functional class was significantly different between the groups (2.9 ± 0.5 vs. 2.2 ± 0.5 in the HF-rEF and in the HF-pEF group, respectively). No differences between the groups were detected with regards to age, plasma glucose levels, total cholesterol, LDL-cholesterol, and triglycerides, while both systolic and diastolic BP values were significantly higher in the HF-pEF group. HF-rEF patients showed higher creatinine and uric acid levels and lower e-GFR and HDL-cholesterol values. No differences were observed in the red blood cell counts between the groups, while a small, but significant, difference was observed in hemoglobin levels (12.3 ± 1.4 vs. 13.6 ± 1.4 g/dl, in the HF-rEF and in the HF-pEF group, respectively). Clinically relevant, despite similar ferritin and transferrin levels between groups, HF-rEF patients showed a significant reduction in iron values and saturated transferrin iron binding capacity (TIBC) percentage, and higher values of unsaturated iron binding capacity (UIBC) and hepcidin levels.

**Table 1:** Anthropometric, biochemical and hemodynamic characteristics of the whole study population and of the two groups separately.

	**All** **(*n* = 50)**	**HF-rEF** **(*n* = 25)**	**HF-pEF** **(*n* = 25)**	***P***
Gender, m/f	41/9	21/4	20/5	
Age, years	70.7 ± 10.7	70.2 ± 12.8	71.1 ± 8.3	0.439
NYHA class	2.6 ± 0.5	2.9 ± 0.5	2.2 ± 0.5	0.0001
**Etiology**				
– Ischemic/hypertensive	26	12	14	0.829
– Valvular	6	3	3	
– Cardiomyopathy/other	18	10	8	
SBP, mmHg	124 ± 14	118 ± 15	129 ± 10	0.0037
DBP, mmHg	72 ± 8	68 ± 7	76 ± 6	0.0001
Glucose, mg/dl	120.8 ± 25.4	124.8 ± 30.6	116.7 ± 23.2	0.296
Cholesterol, mg/dl	132.8 ± 30.9	130.0 ± 27.9	135.7 ± 34.5	0.523
LDL-Cholesterol, mg/dl	69.6 ± 24.3	67.8 ± 20.1	71.5 ± 28.3	0.455
HDL-Cholesterol, mg/dl	44.1 ± 8.5	40.9 ± 8.1	47.2 ± 7.7	<0.0001
Triglyceride, mg/dl	118.3 ± 64.1	121.1 ± 44.5	115.3 + 33.8	0.606
White blood cells, 10^3^/μl	7.12 ± 1.97	7.49 ± 2.10	6.73 ± 1.81	0.176
Red blood cells, 10^6^/μl	4.75 ± 0.50	4.72 ± 0.51	4.78 ± 0.49	0.673
MCV, fl	84.2 ± 3.6	86.3 ± 3.1	82.0 ± 3.0	0.0001
Hemoglobin, g/dl	12.9 ± 1.5	12.3 ± 1.4	13.6 ± 1.4	0.001
Serum iron, mcg/dl	77.1 ± 38.2	61.4 ± 18.3	93.7 ± 33.7	0.0001
Ferritin, ng/ml	104.1 ± 30.5	100.2 ± 24.7	108.1 ± 36.1	0.371
Transferrin, g/l	2.76 ± 0.52	2.83 ± 0.64	2.68 ± 0.29	0.291
UIBC, mcg/dl	251.6 ± 73.1	272.6 ± 74.9	221.7 ± 61.4	0.011
Saturated TIBC, %	25.3 ± 13.1	20.7 ± 8.4	31.1 ± 15.6	0.005
Hepcidin, ng/ml	4.25 ± 1.30	4.61 ± 0.89	3.28 ± 0.69	0.0001
Creatinine, mg/dl	1.19 ± 0.45	1.34 ± 0.55	1.03 ± 0.25	0.013
e-GFR, ml/min/1.73 m^2^	97.5 ± 40.1	78.1 ± 36.1	118.1 ± 33.9	0.0002
Uric acid, mg/dl	6.4 ± 1.7	6.8 ± 1.7	5.7 ± 1.1	0.009

*DBP, diastolic blood pressure; eGFR, estimated glomerular filtration rate; MCV, mean corpuscular volume; SBP, systolic blood pressure; TIBC, transferrin iron binding capacity; UIBC, unsaturated iron binding capacity*.

**Figure 2:**
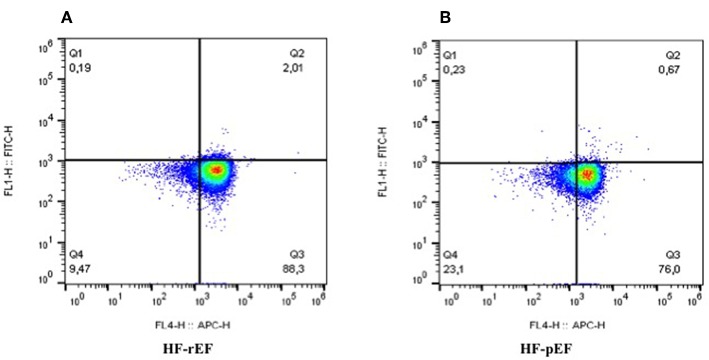
Levels of expression of TLR2 and TLR4 in PBMCs in patients with reduced ejection fraction **(A)** and preserved ejection fraction **(B)**. As evident, **(A)** shows a higher expression of TLRs, in particular of TLR4. FL4-H, TLR4; FL1-H, TLR2; TLRs, toll-like receptors.

In Table 2 we reported the inflammatory parameters of the whole study population and of the two groups separately. HF-rEF patients showed a significant increased expression of both TLR-2 and TLR-4 (Figure 2), as well as significantly raised IL-6, MCP-1, fibrinogen, and hs-CRP values. Notably, even if without statistically significant differences between groups, IL-8 levels resulted beyond the range of normality in both HF-rEF and HF-pEF patients. On the contrary, we detected significantly lower values of IL-10 in the HF-rEF group. NF-κB levels were significantly higher in the HF-rEF group.

**Table 2:** Inflammatory parameters of the whole study population and of the two groups separately.

	**All** ** (*n* = 50)**	**HF-rEF** ** (*n* = 25)**	**HF-pEF** ** (*n* = 25)**	***P***
TLR2, MFI	1.58 ± 0.92	1.90 ± 0.97	1.25 ± 0.76	0.011
TLR4, MFI	3.98 ± 1.56	4.54 ± 1.32	3.38 ± 1.62	0.007
NF-κB	1.80 ± 0.93	2.67 ± 0.60	1.07 ± 0.30	0.0001
IL-6, pg/ml	10.6 ± 7.5	15.4 ± 7.7	6.0 ± 2.8	0.0001
IL-8, pg/ml	20.6 + 10.1	22.4 + 12.8	18.6 + 6.2	NS
IL-10, pg/ml	1.8 ± 1.4	1.4 ± 0.9	2.3 ± 1.7	0.023
MCP-1, pg/ml	451.2 ± 98.1	478.8 ± 81.7	401.4 ± 94.6	0.003
TNF-α, pg/ml	3.5 ± 0.9	3.5 ± 1.2	3.5 ± 0.7	NS
hs-CRP, mg/L	4.0 ± 2.9	5.9 ± 2.6	1.9 ± 1.2	0.0001
Fibrinogen, mg/dl	316.0 ± 73	336 ± 78	285 ± 57	0.011

*hsCRP, High-sensitivity C-reactive protein; IL, Interleukin; MCP1, Monocyte Chemoattractant Protein-1; NF-κB, nuclear factor κB; TLR2, toll-like receptor-2; TLR4, toll-like receptor-4; TNF-α, Tumor necrosis factor alpha*.

**Figure 3:**
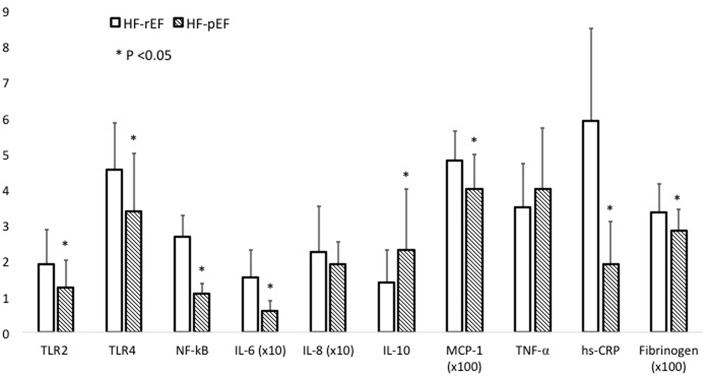
This figure graphically describes inflammatory parameter differences between the HF-rEF and HF-pEF groups. For some parameters the values are expressed as × 10 or × 100, as indicated. Comparisons between groups were made using a student's *t*-test. *N* = 50 for each study group. For Elisa assays samples were tested in duplicate. hsCRP, high-sensitivity C-reactive protein; IL, interleukin; MCP1, monocyte chemoattractant protein-1; NF-κB, nuclear factor kB; TLR, toll-like receptor; TNF-α, tumor necrosis factor alpha.

In Figure 3 we graphically reported differences between groups in inflammatory parameters.

Table 3 describes the echocardiographic characteristics of the whole study population and of the two groups separately. As expected, EF was significantly lower in the HF-rEF group, while i-MVS, d-LVID, s-LVID, LA diameter, i-LAV, E/A ratio, and s-PAP resulted significantly higher in the same group. Similarly, the right ventricular function parameters such as the TAPSe and TAPSe/s-PAP ratio was also significantly impaired in the HF-rEF.

**Table 3:** Echocardiographic parameters of the whole study population and of the two groups separately.

	**All** ** (*n* = 50)**	**HF-rEF** ** (*n* = 25)**	**HF-pEF** ** (*n* = 25)**	***P***
EF, %	41.4 ± 7.8	35.3 ± 3.7	54.1 ± 5.2	0.0001
i-LVM, g/m^2^	150.5 ± 26.1	164.8 ± 21.3	138 ± 23.1	0.0001
EDV/BSA, ml/m^2^	89.4 ± 24.4	103.6 ± 22.3	74.4 ± 15.6	0.0001
ESV/BSA, ml/m^2^	47.6 ± 17.6	63.1 ± 12.3	35.8 ± 10.7	0.0001
i-LAV, ml/m^2^	51.5 ± 13.5	55.4 ± 11.3	47.4 ± 14.3	0.033
E/A	0.9 ± 0.3	1 ± 0.4	0.8 ± 0.1	0.019
E, m/s	0.9 ± 0.3	1.1 ± 0.3	0.9 ± 0.2	0.0001
E/e'	19.5 ± 5.4	18.5 ± 6.8	20.6 ± 3.1	0.166
s-PAP, mmHg	38.5 ± 10.4	41.4 ± 8.7	35.5 ± 11.1	0.042
TAPSe, mm	18.3 ± 3.1	18.1 ± 3.3	18.5 ± 2.6	0.636

*BSA, body surface area; d-LVID, diastolic left ventricular internal diameter; EDV, end-diastolic volume; EF, ejection fraction; ESV, end-systolic volume; i-LAV, indexed left atrium volume; i-LVM, indexed left ventricular mass; s-LVID, systolic left ventricular internal diameter; s-PAP, systolic pulmonary arterial pressure; TAPSe, tricuspid annular plane excursion*.

In Table 4 we reported the correlational analyses between iron status and markers of inflammation and LVEF, hepcidin and markers of inflammation, ferritin and markers of inflammation, and iron status. When considering the correlation between LVEF and iron status, we found a significant and direct correlation with hemoglobin and serum iron in the whole study population and in the HF-rEF group, while in the HF-pEF group the only significant direct correlation was found with serum iron. A significant inverse relationship was found between LVEF and both IL-6 and hs-CRP in the whole study population and in HF-rEF patients; furthermore, in this group we also found an inverse relationship between LVEF and ferritin. The correlational analysis between hepcidin and different parameters showed a significant and direct correlation between IL-6, and TLR4 in the whole study population and in the HF-rEF group, while TLR2 was significantly correlated to hepcidin only in the whole study population. No significant correlations were found for any of the tested parameters in the HF-pEF group. Of note, the correlational analysis between ferritin and different inflammatory and iron status parameters, showed an inverse relationship with both serum iron and hemoglobin, and a direct relationship with IL-6 in the HF-rEF group, thus confirming that, in this group, ferritin levels correlate best with inflammatory parameters rather than with iron status.

**Table 4:** Correlational analyses between different parameters in the whole study population and in the two groups separately.

	**All**	**HF-rEF**	**HF-pEF**
	***R/*P**	***R*/P**	***R*/P**
**LVEF and…**			
Hemoglobin	0.573/0.0001	0.414/0.020	−0.112/0.298
Iron	0.643/0.0001	0.645/0.0001	0.456/0.011
Ferritin	0.043/0.384	−0.594/0.001	0.031/0.443
IL-6	−0.793/0.0001	−0.382/0.030	−0.088/0.337
hs-CRP	−0.772/0.0001	−0.586/0.001	0.075/0.361
**Hepcidin and…**			
IL-6	0.558/ <0.0001	0.398/0.024	0.094/0.377
TLR2	0.244/0.044	0.084/0.345	−0.018/0.466
TLR4	0.346/0.007	0.385/0.029	−0.095/0.326
Ferritin	0.119/0.205	0.229/0.136	0.240/0.124
**Ferritin and…**			
Serum iron	−0.018/ 0.450	−0.413/0.020	−0.022/0.458
Hemoglobin	−0.137/0.171	−0.451/0.012	0.176/0.200
Transferrin	−0.200/0.082	−0.056/0.395	−0.418/0.019
Saturated TIBC %	0.245/0.043	0.216/0.150	0.194/0.177
UIBC	−0.245/0.472	−0.048/0.409	−0.377/0.032
IL-6	0.010/0.433	0.343/ 0.047	0.176/0.200
TLR2	0.024/0.433	0.164/ 0.217	0.039/0.426
TLR4	0.099/0.247	0.188/0.184	0.185/0.187
MCP-1	0.125/0.193	−0.191/0.180	0.446/0.013
IL-8	−0.036/0.402	0.140 /0.253	−0.210/0.157
TNFα	−0.092/0.263	−0.042/0.422	−0.178/0.197
hs- CRP	−0.099/0.248	0.100/0.317	−0.099/0.320
NF-κB	−0.012/0.186	0.065/0.378	0.070/0.370

*hs-CRP, High-sensitivity C-reactive protein; IL, Interleukin; LVEF, left ventricular ejection fraction; MCP1:Monocyte Chemoattractant Protein-1; NF-κB, nuclear factor kB; TIBC, transferrin iron binding capacity; TLR2, toll-like receptor-2; TLR4, toll-like receptor-4; TNF-α, Tumor necrosis factor alpha; UIBC, unsaturated iron binding capacity*.

## Discussion

There is convincing evidence that inflammation is associated with an increased CV risk, independent of traditional risk factors, even if clinical trials designed to test the efficacy of anti-inflammatory therapy in the reduction of CV outcomes gave conflicting results (19, 20). Even if some inflammatory factors have been found to be elevated in failing patients, the relationship between inflammation and HF still remains to be completely elucidated. It is well demonstrated that one of the putative pathogenetic underlying mechanisms of this inflammatory status is recognized as the activation of the immune system, both innate and adaptive (11, 21–24). Of interest, some evidence showed that HF functional class deterioration is related to an increase of some of these inflammatory factors (23). In keeping with this, our study demonstrates, for the first time, different immune-mediated mechanisms of inflammation in HF patients with reduced or preserved EF. In fact, we observed a significant increase of pro-inflammatory cytokines and a parallel decrease of the protective cytokine IL-10 in HF-rEF patients, reflecting a consensual increased activation of TLRs. Our data are confirmatory of previously published evidence demonstrating an impairment in left ventricular function attributable to chronic TLR-2 and TLR-4 chronic activation (11, 21–25). Obviously, these findings reflect the dissimilar morphological and functional cardiac impairment characterizing the two different forms of HF, as a consequence of both dissimilar TLRs expression and oxidative stress levels detected.

Moreover, inflammatory burden, *per se*, causes activation of the transcriptional factor NF-κB that promotes the overexpression of some proinflammatory cytokines, as observed in our population, initiating a vicious cycle that, if not interrupted, leads to cardiac end-stage disease. In fact, our patients with reduced EF showed almost doubled values of NF-κB. It is important to remark that, even if not investigated in our study, proinflammatory mediators and oxidative stress induce both dysfunctional mitochondrial autophagy and fibroblast activation and proliferation with increased myocardial fibrosis, which contributes to the progression of cardiac dysfunction (23). It is worth noting that in the HF-rEF group, we observed a significant increase of MCP-1, which is involved in early post-myocardial infarction ventricular remodeling (21).

Another relevant result of this study is the different iron statuses, observed for the first time, in patients with HF-rEF and HR-pEF, respectively. Particularly, we documented that HF-rEF patients have a significant reduction in serum iron concentration, even if in the normal range, and a saturated TIBC percentage, while UIBC was significantly higher, in absence of overt anemia. The underlying pathogenetic mechanism may be recognized in the different hepcidin biological activities exerted in HF-rEF and HR-pEF patients. In fact, of clinical relevance, we found increased hepcidin levels in HF-rEF that, probably, induces a higher inhibition of duodenal iron absorption and its mobilization from storage sites. It is well established that hepcidin levels increase under an inflammatory stimulus, as also documented in our study on the increase of some pro-inflammatory cytokines. As a consequence of this inflammatory activation, in the HF-rEF subjects we also observed a significant increase in both hs-CRP and fibrinogen levels that are acute phase response inflammatory proteins, and that are routinely available in clinical practice. Furthermore, we found significantly reduced hemoglobin concentrations in the HF-rEF group; this finding may be attributable to several causes, such as hemodilution, iron deficiency, or both.

In accordance with Van Linthout (26), our findings do not allow establishing if inflammation has a causative or a consequential role in HF; in fact, inflammation and HF are strongly associated, activating some reverberant circuits that reinforce each other. In keeping with this, HF-associated comorbidities such as hypertension, chronic obstructive pulmonary disease, type-2 diabetes mellitus, obesity, atherosclerosis etc., are all characterized by mild inflammation and increased oxidative stress (27, 28). Even if this inflammation starts with traditional CV risk factors, it is important to underline that this inflammatory burden is also associated with the activation of some neurohormonal systems, such as the renin-angiotensin-aldosterone system (29). This activation, in turn, induces an increase in renal sodium and water reabsorption promoting blood volume expansion, and an increase of both cardiac post-load and cardiac work; in addition, the associated increased aortic impedance and peripheral vascular resistances also negatively impact cardiac function deterioration.

Consistent with this evidence, pharmacological treatment of HF continuously evolves in order to improve both survival and quality of life. In fact, a one-size-fits-all approach may not be appropriate for all patients with HF, and novel drugs have been proposed for HF treatment. Among these, evidence has demonstrated that intravenous iron supplementation in HF-rEF patients with absolute or relative ID, with or without anemia, is capable of improving cardiac function (8–10). These results confirm the detrimental effect of ID on cardiac contractility of failing patients, particularly for those with HF-rEF, probably as a consequence of mitochondrial impairment that may further promote cardiomyocytes dysfunction (30–33). However, even if our HF-pEF patients did not exhibit ID, they already have an inflammatory burden that, if not recognized and treated early, could accelerate disease progression toward HF-rEF. The effects of iron supplementation on the innate immunity-driven inflammatory cascade have not yet been fully elucidated, nor does our data contribute to reaching a possible explanation. For this reason, further investigations are needed.

In conclusion, our data contribute to further elucidate pathophysiologic mechanisms leading to cardiac dysfunction, and consequently consent to hypothesize new therapeutic targets for congestive HF.

### Study Limitations

Our study has some limitations. First, due to the small sample size, our findings need to be confirmed in wider studies. Related to this point, our patients were very heterogeneous in terms of HF etiology; this can be regarded a limitation since the underlying causes of inflammation are not similar, but can also be regarded a strength because our population is representative of a real-life setting. Second, we evaluated neither intracellular iron nor atrial natriuretic peptides, that can potentially add important information. Finally, since we did not administer iron supplementation in patients with ID, we do not know the effects of iron therapy on both inflammatory parameters and cardiac function in our study population.

## Data Availability Statement

The datasets generated for this study are available on request to the corresponding author.

## Ethics Statement

The studies involving human participants were reviewed and approved by Comitato Etico Sezione Calabria Centro. The patients/participants provided their written informed consent to participate in this study.

## Author Contributions

MP and FP contributed to the design of the study and approved the final manuscript. RZ, MH, and SC drafted the manuscript. SM, AP, ES, SG, and MG contributed to data collection. GS revised the manuscript.

### Conflict of Interest

The authors declare that the research was conducted in the absence of any commercial or financial relationships that could be construed as a potential conflict of interest.
